# Curcuma DMSO extracts and curcumin exhibit an anti-inflammatory and anti-catabolic effect on human intervertebral disc cells, possibly by influencing TLR2 expression and JNK activity

**DOI:** 10.1186/1476-9255-9-29

**Published:** 2012-08-21

**Authors:** Marina Klawitter, Lilian Quero, Juergen Klasen, Alexia N Gloess, Babette Klopprogge, Oliver Hausmann, Norbert Boos, Karin Wuertz

**Affiliations:** 1 Spine Research Group, Competence Center for Applied Biotechnology and Molecular Medicine, University of Zurich, Zurich, Switzerland; 2 University Hospital Balgrist, University of Zurich, Zurich, Switzerland; 3 Institute of Chemistry and Biological Chemistry, Zurich University of Applied Sciences, Waedenswil, Switzerland; 4 Department of Neurosurgery, Clinic St. Anna, Lucerne, Switzerland; 5 AOSpine Research Network, Duebendorf, Switzerland; 6 Institute of Physiology and Zurich Center for Integrative Human Physiology (ZIHP), University of Zurich, Zurich, Switzerland; 7 Institute for Biomechanics, Swiss Federal Institute of Technology (ETH), Zurich, Switzerland; 8 Bone and Stem Cell Research Group, Competence Center for Applied Biotechnology and Molecular Medicine, University of Zurich, Zurich, Switzerland; 9 Institute for Biomechanics, Swiss Federal Institute of Technology (ETH) Zurich, Schafmattstrasse 30, 8093, Zurich, Switzerland

**Keywords:** Human intervertebral disc cells, Curcumin, Curcuma, Proinflammatory cytokines, Matrix degrading enzymes, NF-κB, Toll-like receptors, MAP kinase, Back pain, HPLC/MS

## Abstract

**Background:**

As proinflammatory cytokines seem to play a role in discogenic back pain, substances exhibiting anti-inflammatory effects on intervertebral disc cells may be used as minimal-invasive therapeutics for intradiscal/epidural injection. The purpose of this study was to investigate the anti-inflammatory and anti-catabolic potential of curcuma, which has been used in the Indian Ayurvedic medicine to treat multiple ailments for a long time.

**Methods:**

Human disc cells were treated with IL-1β to induce an inflammatory/catabolic cascade. Different extracts of curcuma as well as curcumin (= a component selected based on results with curcuma extracts and HPLC/MS analysis) were tested for their ability to reduce mRNA expression of proinflammatory cytokines and matrix degrading enzymes after 6 hours (real-time RT-PCR), followed by analysis of typical inflammatory signaling mechanisms such as NF-κB (Western Blot, Transcription Factor Assay), MAP kinases (Western Blot) and Toll-like receptors (real-time RT-PCR). Quantitative data was statistically analyzed using a Mann Whitney *U* test with a significance level of p < 0.05 (two-tailed).

**Results:**

Results indicate that the curcuma DMSO extract significantly reduced levels of IL-6, MMP1, MMP3 and MMP13. The DMSO-soluble component curcumin, whose occurrence within the DMSO extract was verified by HPLC/MS, reduced levels of IL-1β, IL-6, IL-8, MMP1, MMP3 and MMP13 and both caused an up-regulation of TNF-α. Pathway analysis indicated that curcumin did not show involvement of NF-κB, but down-regulated TLR2 expression and inhibited the MAP kinase JNK while activating p38 and ERK.

**Conclusions:**

Based on its anti-inflammatory and anti-catabolic effects, intradiscal injection of curcumin may be an attractive treatment alternative. However, whether the anti-inflammatory properties *in vitro* lead to analgesia *in vivo* will need to be confirmed in an appropriate animal model.

## Background

Degeneration of the intervertebral disc is characterized by enhanced proteolytic degradation of extracellular matrix proteins as well as altered matrix protein synthesis. This overall catabolic shift leads to changes in the tissue structure that have been extensively described in the literature [1-7]. Although large structural changes can be observed during degeneration, this age-related process does not necessarily cause pain symptoms.

There is certain evidence in the literature that in a subgroup of patients, painful disc degeneration is characterized by increased levels of proinflammatory cytokines, e.g. interleukin 1β (IL-1β), interleukin 6 (IL-6), interleukin 8 (IL-8) and tumor necrosis factor α (TNF-α) [6,8-11]. Although proinflammatory mediators seem to play a crucial role in intervertebral disc diseases, little is known about inflammatory pathways in intervertebral disc cells. Results from studies on the pathogenesis of cartilage degeneration indicate that proinflammatory processes are mostly regulated by the transcription factor NF-κB [12], whose activity is tightly regulated *in vivo*, e.g. by activation of the so-called Toll-like receptors (TLRs) [13]. Another important inflammatory pathway is the MAP kinase pathway that consists of a family of protein kinases with the major members being p38, ERK and JNK [14,15]. Due to the lack of knowledge concerning the molecular events underlying discogenic back pain, treatment of painful disc disease is currently limited, with typical options for the patient being conservative treatment (e.g. physiotherapy) and oral pain medication, both of which often only have a temporary effect. Other options are various types of surgical interventions, but these lead to high risks for the patients and high costs for the health care systems. Therefore, research in the most recent past has concentrated on the development of minimal-invasive, yet effective new treatment options, covering approaches from cell and gene therapy to anti-inflammatory substances for intradiscal injection. Currently, corticosteroidal substances are frequently used, which are known to have a significant risk for side effects and may cause disc space infections [16]. Although research on biodrugs with regard to spinal diseases is yet rare, these novel anti-inflammatory candidates could potentially benefit patients with discogenic back pain.

Curcuma (C*urcuma longa* L., Zingiberaceae) is a perennial herb that is cultivated in Asian countries. As a powder, it has not only been used for cooking for centuries, but also as a drug in the traditional Chinese and Indian medicine, treating e.g. diabetic wounds, hepatic disorders, rheumatism and sinusitis [17]. Numerous publications demonstrated an anti-inflammatory effect of curcuma, with its effect probably being related to a class of substances called curcuminoids [18].

Based on a thorough literature review, we hypothesize that curcuma has the potential to interfere with catabolic and inflammatory pathways. Hence, the aim of this study was to analyze the effects of curcuma extracts as well as of one selected component of curcuma on IL-1β mediated cellular responses of human intervertebral disc cells *in vitro*. Additionally, its mechanism of action was investigated by testing for involvement of NF-κB, MAP kinases (i.e. p38, ERK, JNK) and TLR2.

## Methods

### General experimental design

As an inflammatory environment is thought to be present in (a subgroup of) patients with discogenic back pain, human intervertebral disc cells (cultured in 2D) were pretreated with recombinant IL-1β, thus increasing the levels of proinflammatory cytokines and matrix degrading enzymes. Thereafter, different solvents (DMSO, ethanol) were used to prepare sequential curcuma extracts and tested for their ability to reduce inflammatory and catabolic gene expression after 6 hours. The presumably most abundant bioactive substance in the most potent extract was chosen based on structure-based solubility, information in the literature and identification using HPLC/MS analysis (i.e. curcumin) and tested in the same setting, using various concentrations. A mechanistic investigation, looking at involvement of the NF-κB, MAP kinase and TLR2 pathway, was performed for curcumin as well.

### Human intervertebral disc cell culture

Human intervertebral disc tissue (nucleus pulposus and annulus fibrosus) was removed from 27 patients undergoing spinal surgery for discectomy or interbody fusion for degenerative disc disease or disc herniation (for detailed information see Table 1). Informed consent was obtained from all patients prior to surgery in accordance with the institutional review board.

**Table 1:** Demographic data on surgical disc samples (M = male; F = female)

**Nr.**	**Sex**	**Age**	**Level**	**Pathology**	**Grade**	**Experiments**
1	F	15	L5/S1	Herniation	4	G
2	M	35	L4/5	Herniation	4	G
3	M	46	L3/4	Herniation	3	G
4	M	60	L4/5	Herniation	4	G
5	F	56	L1/2	Herniation	5	G
6	F	48	L4/5	Herniation	4	G
7	M	59	L5/S1	DDD	4	G
8	M	39	L4/5	Herniation	3	G
9	M	60	L4/5	Herniation	3	G
10	F	51	L4/5	DDD	4	G
11	F	48	L4/5	DDD	3	G
12	M	59	L5/S1	Herniation	3	G
13	F	42	L4/5	Herniation	4	G
14	M	28	L5/S1	Herniation	3	V
15	F	71	L1/2	Herniation	5	V
16	F	46	L5/S1	Herniation	5	V
17	M	61	L4/5	Herniation	3	V
18	F	59	L4/5	Herniation	3	V
19	M	57	L4/5	Herniation	4	P
20	M	48	L4/5	Herniation	3	P
21	F	23	L5/S1	Herniation	3	P
22	F	43	L4/5	Herniation	4	P
23	M	49	L5/S1	Herniation	4	P
24	F	50	L5/S1	Herniation	3	P
25	F	45	L5/S1	Herniation	4	P
26	F	40	L4/5	Herniation	4	P
27	N	26	L4/5	Herniation	4	P

Grade = Pfirrmann Grading; Pathology: DDD = Degenerative disc disease Experiments: V = Viability, G = Gene expression analysis, P = Pathway analysis.

Intervertebral disc cells were released from the tissue by enzymatic digestion with 0.2% collagenase NB4 (Serva, Germany) and 0.3% dispase II (Roche Diagnostics, Switzerland) in PBS (37°C, 5% CO_2_) for approximately 4 hours. After digestion, the tissue suspension was filtered (70 μm cell strainer, BD Biosciences, Belgium), washed and cells were seeded and expanded in DMEM/F12 (Sigma, Switzerland) supplemented with 10% FCS, penicillin (50 units/ml), streptomycin (50 μg/ml) and ampicillin (125 ng/ml) (all Invitrogen, Germany), with medium changes once to twice a week and expansion up to passage 2 or 3.

### Preparation of curcuma extracts

Organic curcuma from McCormick (Promena, Switzerland) was used to prepare sequential DMSO and ethanol extracts. Briefly, curcuma was dispersed in DMSO at a concentration of 320 mg/ml, incubated on the shaker at room temperature for 10 min and centrifuged at 2000 rpm for 10 min before taking off the DMSO fraction. The remaining pellet was then dispersed in 100% ethanol and the procedure was repeated. After removal of the ethanol fraction, the thereafter remaining pellet was discarded. For each experiment, the fractions were prepared freshly in order to avoid any damage due to freezing/thawing.

### HPLC/MS analysis of the curcuma DMSO and EtOH extracts

The DMSO and EtOH extracts of curcuma were analysed by high performance liquid chromatography (1200 Series HPLC, Agilent), coupled to a mass spectrometer (6130 series MS, Agilent). The chromatography of the curcuma extracts was performed according to Wichitnithad *et al.*[19], using a RP-C18 column (Agilent Eclipse Plus, 100 mm × 2.1 mm i.d., 1.8 μm). For identification of the curcuminoids, measurements were carried out with a multimode source (electrospray **(**ESI) ionization mode: positive mode; drying gas flow: 12 l/min; drying gas temperature: 350°C; nebulizer pressure: 50 psig; fragmentor voltage: 70 V; capillary voltage: 4000 V). The quantification of the most abundant curcuminoids was done at a wavelength of 425 nm, with commercially available curcumin (Sigma Aldrich) as an external standard.

### Viability measurement

Cells seeded in 24 well plates were treated with different concentrations of curcuma DMSO extract (25, 50, 100, 250, 500 or 1000 μg/ml), curcuma ethanol extract (25, 50, 100, 250, 500 or 1000 μg/ml) or curcumin (1, 5, 10, 20, 50 or 100 μM - dissolved in DMSO). All experiments were performed in triplicates on cells from 5 independent biopsies. After 6, 18 and 30 hours, toxicity was analyzed using the MTT assay: A fresh sterile solution of MTT [3-(4,5-Dimethylthiazol-2-yl)-2,5-diphenyl tetrazolium bromide] (Sigma, Switzerland) with a concentration of 0.5 mg/ml in DMEM/F12 was prepared, 500 μl were added to each well and incubated for 4 hours at 37°C. MTT was discarded, cells were lysed with DMSO for 5 min at 37°C and absorbance was measured at 565 nm. Absorbance of treated cells was calculated relative to absorbance of untreated control cells, which was set to 100% (only changes in viability of >10% were considered). Concentrations that were non-toxic even at late time points were chosen for subsequent experiments. Results of the MTT assay were previously shown to be comparable to other viability measurement techniques (DNA content by Picogreen assay; cell counting) [20].

### Gene expression analysis

Human intervertebral disc cells were serum starved for 2 hours and then exposed to 5 ng/ml IL-1β (Peprotech, Great Britain) for 2 hours before adding 100 μg/ml curcuma DMSO extract or 100 μg/ml curcuma EtOH extract for 6 hours. Untreated control cells were included to verify the inflammatory and catabolic response induced by IL-1β treatment. As we were able to show that the solvents did not influence cellular behavior (see Additional file 1: Figure 3 and Additional file 2: Figure 4), all groups were treated with the respective volume (0.03%) of either DMSO or EtOH in all experiments. Therefore, changes in gene expression are either calculated relative to controls (+0.03% DMSO or EtOH) or relative to IL-1β prestimulated cells (+0.03% DMSO or EtOH). Based on the results with curcuma extracts and data obtained by HPLC/MS analysis, a 25 mM stock solution of curcumin (which is one of the major DMSO-soluble, bioactive components of curcuma) was prepared and cells were treated with final concentrations of 5, 10 or 20 μM curcumin for 6 hours after IL-1β prestimulation. Taking the approximate percentage of curcumin in curcuma powder (~2%) into account, the applied range of curcumin (5–20 μM) was predicted to be similar to the final concentration of curcumin when using the above mentioned curcuma extracts (100 μg/ml). All gene expression experiments were performed on cells from five independent biopsies.

After treatment, cells were harvested by trypsin treatment and total RNA was isolated using the PureLink RNA Mini Kit (Ambion, Switzerland) according to the manufacturer’s instructions. cDNA was synthesized using TaqMan Reverse Transcription Reagents (Applied Biosystems, Switzerland) and gene expression of IL-1β, IL-6, IL-8, TNF-α, MMP1, MMP3, MMP13, TLR2 and TBP (TATA box binding protein = housekeeping gene) was analyzed. Human specific probes and primers (Applied Biosystems, Switzerland, see Table 2), TaqMan real-time RT-PCR Mix (Applied Biosystems, Switzerland) and 10–30 ng of cDNA (depending on the expression level of the respective gene) were mixed and measured in duplicates using the StepOne Plus Real-Time PCR System (Applied Biosystems, Switzerland). The comparative ct method (= 2^-ΔΔCt^ method) [21] was used to quantify PCR data. In order to calculate changes in gene expression induced by curcuma/curcumin, gene expression in IL-1β-treated cells was set to 100% and gene expression of IL-1β/curcuma or IL-1β-/curcumin-treated cells was calculated relative to IL-1β-treated cells (containing the respective amount of either DMSO or EtOH as well).

**Table 2:** Primers/Probes used for real-time RT-PCR (TaqMan® Gene Expression Assays, Applied Biosystems)

**Gene**	**Primer sequence number**	**Base pairs**
Interleukin-1β (IL-1β)	Hs00174097_m1	94
Interleukin-6 (IL-6)	Hs00174131_m1	95
Interleukin-8 (IL-8)	Hs00174103_m1	101
Matrixmetalloproteinase-1 (MMP1)	Hs00233958_m1	133
Matrixmetalloproteinase-3 (MMP3)	Hs00968308_m1	98
Matrixmetalloproteinase-13 (MMP13)	Hs00233992_m1	91
TATA box binding protein (TBP)	Hs00427620_m1	91
Toll-like receptor 2 (TLR2)	Hs00152932_m1	80
Tumor necrosis factor alpha (TNF-α)	Hs00174128_m1	80

Data was obtained by real-time RT-PCR (ΔΔct method) (n = 10).

### Western Blot for NF-κB (p65)

In order to investigate whether changes in NF-κB/p65 translocation occur after treatment with curcumin (substance with most prominent effects, see results), disc cell cultures were either kept untreated, treated with 5 ng/ml IL-1β alone or co-treated with 20 μM curcumin for 60 min.

Nuclear extracts were prepared by washing trypsin-harvested cells with 10 mM HEPES (pH 7.9), containing 1.5 mM MgCl_2_, 10 mM KCl, 1 mM PMSF, 5 mM DTT and 0.1% protease inhibitors (pepstatin-A, leupeptin and bestatin). Then, cells were lysed with 0.1% NP-40 for 5 min, centrifuged for 5 min at 10’000 rpm (4°C) and supernatants were discarded. Nuclear pellets were washed with 0.1% NP-40 and lysed for 20 min with 20 mM HEPES (pH 7.9), containing 1.5 mM MgCl_2_, 420 mM NaCl, 25% glycerol, 1 mM PMSF and 5 mM DTT as well as protease inhibitors (see above). After centrifugation, protein content was measured by Bradford assay (Bio-rad, Germany).

Nuclear extracts of untreated, IL-1β-treated and IL-1β/curcumin-treated cells were separated on a SDS-polyacrylamid gel and transferred to a PVDF membrane (Amersham, Switzerland). The membrane was incubated with a p65 antibody (Santa Cruz, Germany) followed by incubation with an appropriate HRP secondary antibody before analyzing chemiluminescence. PARP (Poly [ADP-ribose] polymerase) was used as a loading control. The assay was performed on cells from three independent biopsies.

### Transcription factor assay for NF-κB (p65)

In order to detect specific NF-κB DNA binding activity in nuclear extracts, the NF-κB (p65) Transcription Factor Assay (Cayman, Estonia) was used according to the protocol provided by the manufacturer. Briefly, a specific double stranded DNA (dsDNA) sequence containing the NF-κB response element was immobilized to the wells of a 96 well plate. Nuclear extracts were prepared as described above and added to the coated wells. NF-κB contained in the added nuclear extract bound specifically to the NF-κB response element and was detected by addition of the provided specific primary antibody directed against NF-κB (p65). A secondary antibody conjugated with HRP was added, a colorimetric readout at 655 nm was performed and data was quantified as indicated in the protocol. The assay was performed on cells from two independent biopsies.

### Western blot for MAP kinases (p38, ERK, JNK)

Whole cell extracts of untreated, IL-1β-treated and IL-1β/curcumin-treated cells were prepared after 15 min (using standard protocols) to investigate whether curcumin acts on typical MAP kinases. Protein content was measured by Bradford assay and immunoblotting of whole cell extracts was performed as described for p65, but membranes were incubated with antibodies recognizing either unphosphorylated or phosphorylated p38, ERK (p42/44) or JNK (Cell Signaling, USA) before adding an HRP-labeled rabbit secondary antibody and analyzing chemiluminescence. Tubulin was used as a loading control. The assay was performed on samples from five independent experiments.

### Statistical analysis

All quantitative data (cytotoxicity, gene expression) was statistically analyzed using a Mann Whitney *U* test on the SPSS statistics software and differences were considered statistically significant at p < 0.05 (two-tailed).

## Results

### Cytotoxicity of curcuma extracts and curcumin

**Figure 1:**
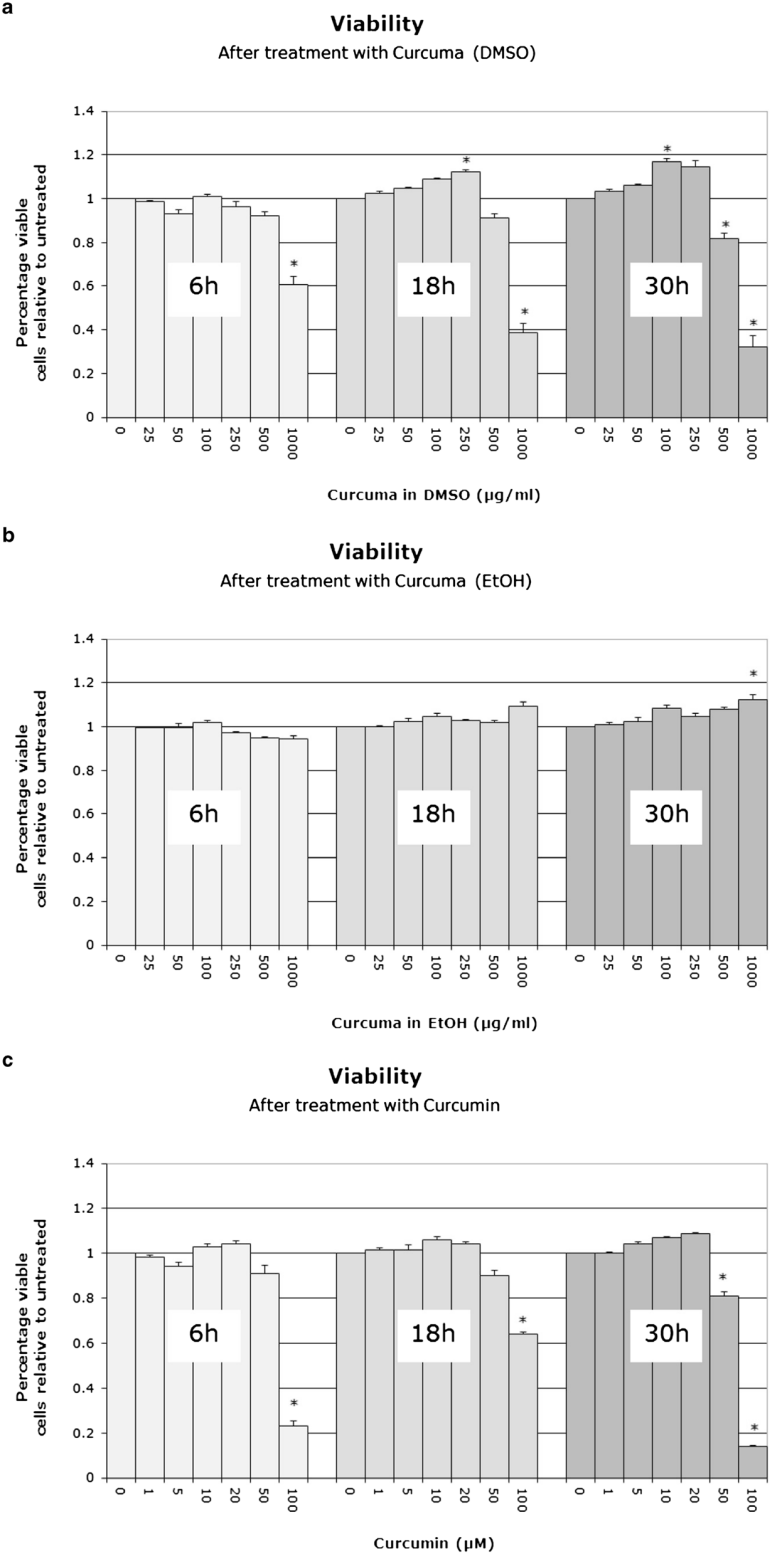
**Cytotoxicity of the curcuma DMSO extract (1a), curcuma ethanol extract (1b) and curcumin (1c) after 6, 18 and 30 hours.** Data was obtained by use of the MTT assay and is presented as Mean and SEM (n = 5). Asterisks indicate statistical significance (p < 0.05).

Cytotoxicity of curcuma extracts (DMSO, ethanol) and curcumin was determined after 6, 18 and 30 hours (i.e. toxicity for short and long time points) using the MTT assay. For the curcuma DMSO extract, cell viability was constricted at concentrations of 500 μg/ml (30 hours) and 1000 μg/ml (all time points) (Figure 1a). A slight proliferative effect was observed for 100 μg/ml (30 hours) and 250 μg/ml (18 hours). For the curcuma ethanol extract, no cytotoxic effect could be observed at any time point up to a concentration of 1000 μg/ml (Figure 1b). For curcumin, cytotoxic effects could be observed at concentrations of 50 μM (30 hours) and 100 μM (all time points) (Figure 1c).

### Changes in gene expression with IL-1β prestimulation

With IL-1β treatment, we could observe a significant increase in the mRNA levels of all genes of interest at the time of analysis (6 hours). Data for all genes is shown in Table 3 as mean, SEM and p-value (data based on analysis of cells from 10 independent biopsies).

**Table 3:** Effects of IL-1β stimulation on mRNA levels of candidate genes after 6 hours

**Gene**	**Mean (Fold Change)**	**SEM**	**p-Value**
IL-1β	246.34	48.12	<0.001
IL-6	2895.17	1571.28	<0.001
IL-8	241.89	56.71	<0.001
MMP1	166.05	31.06	<0.001
MMP3	260.58	70.75	<0.001
MMP13	119.06	29.49	<0.001
TNF-α	21.45	7.16	<0.001
TLR2	8.49	1.64	<0.001

Data was obtained by real-time RT-PCR (ΔΔct method) (n = 10).

### Changes in gene expression with curcuma DMSO and ethanol extracts

As shown in the Supplementary Material (Additional file 1: Figure 3 and Additional file 2: Figure 4), neither DMSO nor EtOH at the used concentration (0.03%) influenced the expression of the inflammatory and catabolic target genes.

**Figure 2:**
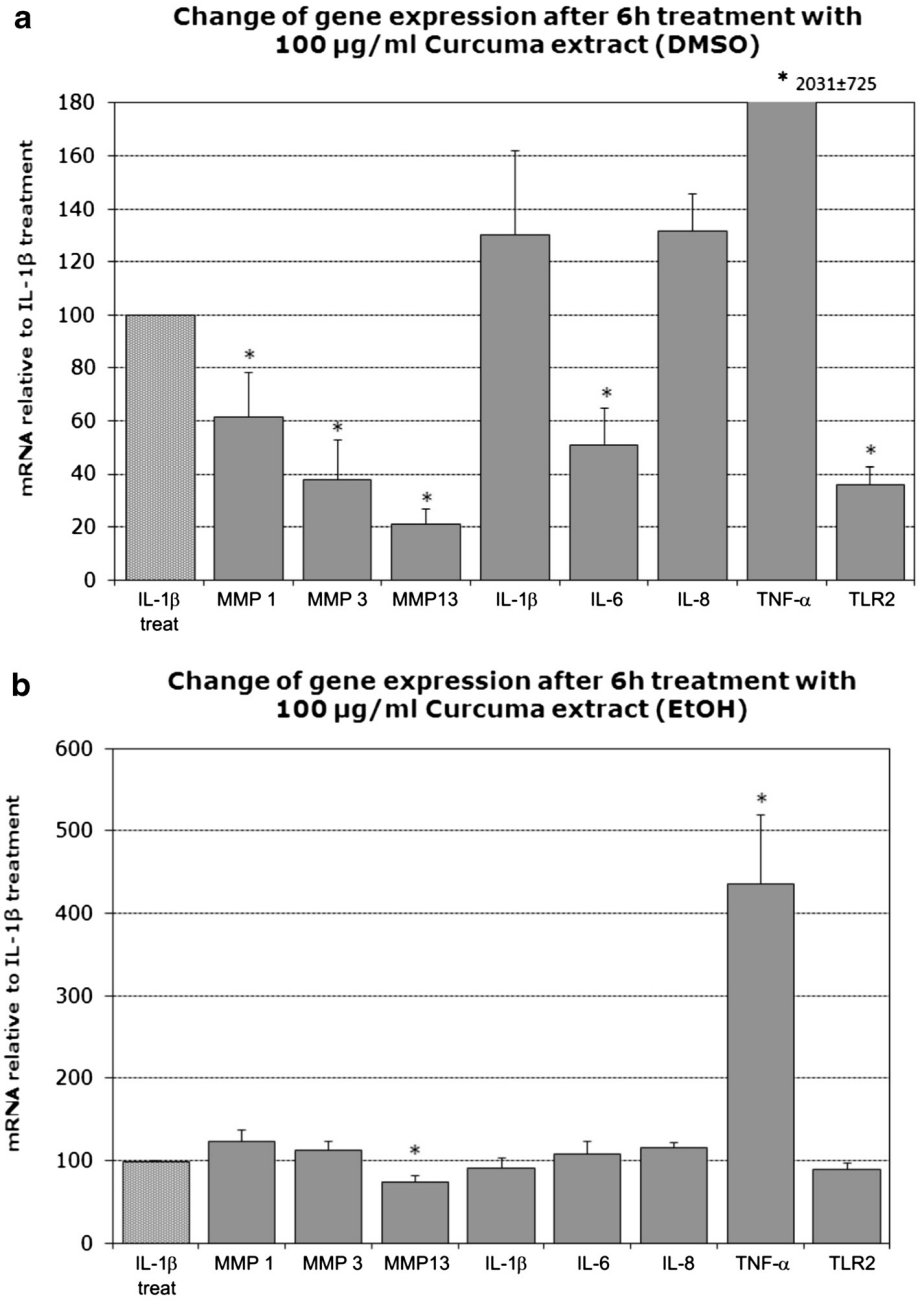
**Effects of the curcuma DMSO extract (2a) and curcuma ethanol extract (2b) on mRNA levels of candidate genes after 6 hours, indicated as fold change relative to IL-1β-treatment (IL-1β treated cells also contain 0.03%of DMSO or EtOH respectively).** Data was obtained by real-time RT-PCR (2^-ΔΔCt^ method) and is presented as Mean and SEM (n = 5). Asterisks indicate statistical significance (p < 0.05). Each gene was normalized to its respective IL-1β treatment (IL-1β + solvent), which was always set to 100% (only one exemplary control bar is shown).

Treatment with the curcuma DMSO extract resulted in a significant inhibition of MMP1, MMP3 and MMP13 after 6 hours, relative to IL-1β prestimulated cells (which are also supplemented with the respective volume of DMSO). While no changes occurred in the expression of IL-1β and IL-8, a significant inhibition of IL-6 was observed. However, we noticed a strong induction of TNF-α expression at this early time point. Expression of TLR2 was significantly reduced. For all results see Figure 2a as well as Additional file 3: Table 1 for summarized values.

Compared to IL-1β prestimulated cells, treatment with the curcuma EtOH extract did not cause any changes in gene expression after 6 hours for MMP1 and MMP3 while slightly decreasing MMP13 expression. Expression of IL-1β, IL-6 and IL-8 also remained unchanged, but TNF-α expression was increased. TLR2 expression was not influenced. For all results see Figure 2b as well as Additional file 3: Table 1 for summarized values.

### Analysis of the curcuma DMSO and EtOH extracts (HPLC/MS)

**Figure 3:**
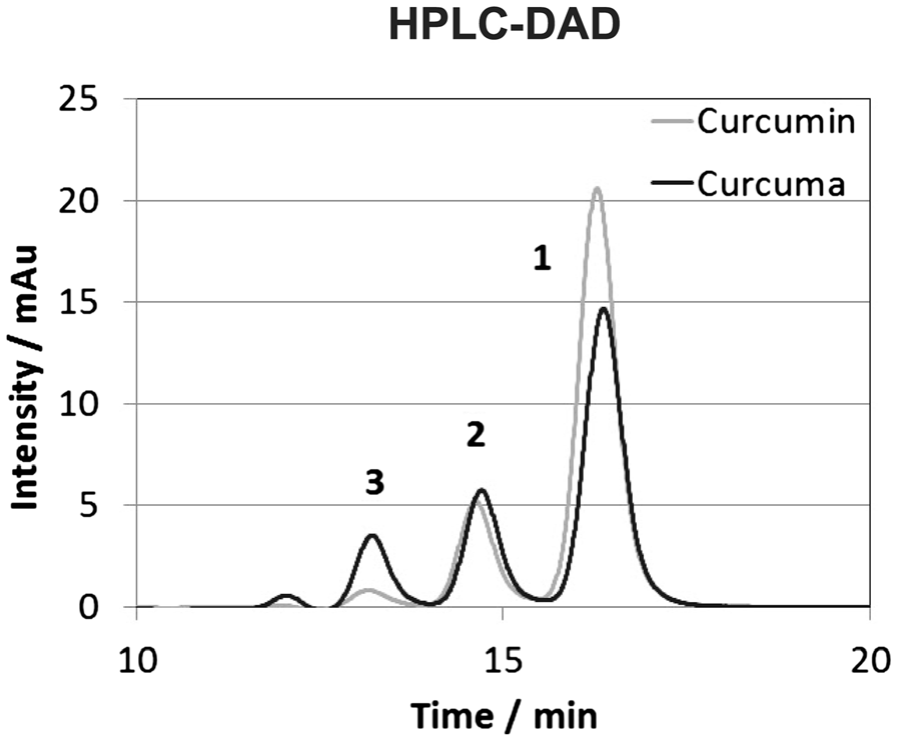
**Analysis of the composition of the curcuma DMSO extract (black line) and the reference substance curcumin (grey line, >94% purity with demethoxycurcumin and bisdemethoxycurcumin as impurity) in DMSO by HPLC-DAD (detection wavelength 425 nm).** The three peaks were identified as curcumin (1, retention time 16.9 min), demethoxycurcumin (2, retention time 15.4 min) and bisdemethoxycurcumin (3, retention time 13.7 min) by their (M + H)^+^ ions in the HPLC/MS analysis.

Based on the above shown results, the DMSO fraction seemed to contain one or more anti-catabolic and anti-inflammatory substances. Taking the solubility of the various components of curcuma as well as the literature-based preselection of anti-inflammatory components of curcuma into account, the curcuminoid curcumin was chosen to be the most promising candidate substance with biological activity. In order to proof that curcumin was indeed present in the DMSO extract, HPLC/MS analysis was performed on the stock extracts (320 mg/ml). The results showed that predominantly curcumin (6.32 mg/ml) (Peak 1) was present in the extract (retention time 16.9 min, (M + H)^+^ at m/z 369.1), followed by its precursors demethoxycurcumin (retention time 15.4 min, (M + H)^+^ at m/z 339.1) (Peak 2) and bisdemethoxycurcumin (retention time 13.7 min, (M + H)^+^ at m/z 309.1) (Peak 3) and other unidentified compounds with little absorbance (Figure 3).

**Figure 4:**
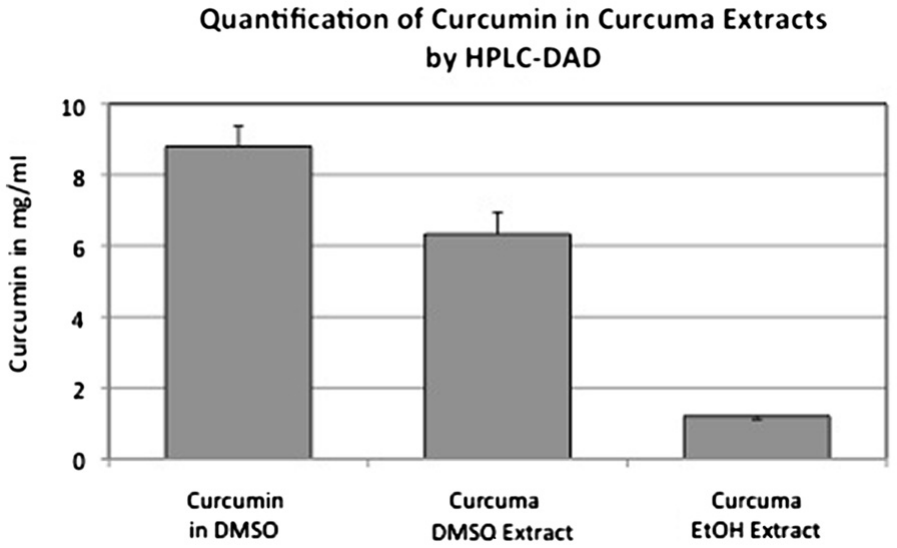
**HPLC/MS measurement of the curcumin concentration in curcuma DMSO and curcuma EtOH stock extracts (320 mg/ml), prepared by the described sequential extraction procedure.** In addition, the curcumin stock solution (25 mM; in DMSO) was quantified.

As curcumin is also soluble in EtOH, we performed a sequential extraction process described under Materials and Methods in order to aggregate curcumin in the DMSO extract. Both, the sequential EtOH extract (stock extract = 320 mg/ml) as well as the pure curcumin stock solution in DMSO (25 mM) were also measured by HPLC/MS. While the curcuma DMSO extract contained 6.32 mg/ml of curcumin, the sequential curcuma EtOH extract contained only 1.2 mg/ml (Figure 4). Curcumin itself showed the highest value and was in a similar range as the curcuma DMSO extract (8.80 mg/ml) (Figure 4).

### Changes in gene expression with curcumin

**Figure 5:**
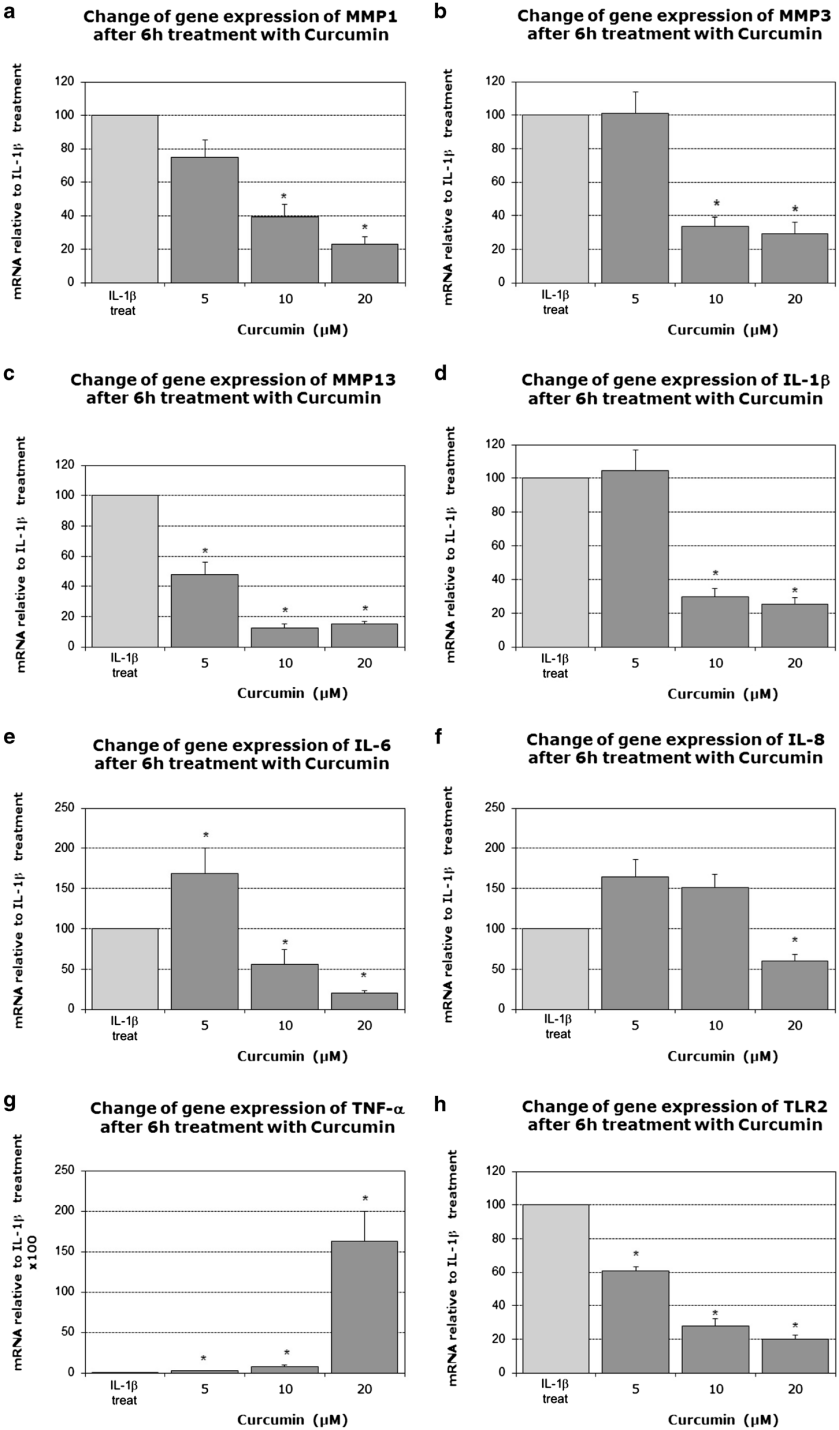
**Effects of curcumin (5, 10 or 20 μM) on mRNA levels of candidate genes after 6 hours, indicated as fold change relative to IL-1β-treatment: MMP1 (5a); MMP3 (5b); MMP13 (5c); Interleukin-1β (5d); Interleukin-6 (5e); Interleukin-8 (5f); TNF-α (5g); TLR2 (5h) (IL-1β treated cells also contain 0.03% of DMSO).** Data was obtained by real-time RT-PCR (2^-ΔΔCt^ method) and is presented as Mean and SEM (n = 5). Asterisks indicate statistical significance (p < 0.05). Each gene was normalized to its respective IL-1β treatment (IL-1β + DMSO), which was always set to 100% (only one exemplary control bar is shown).

Based on the above-mentioned findings, curcumin was investigated at different concentrations (equalizing to the approximate concentration of curcumin in the curcuma extract) in more detail at the 6 hour time point. Treatment with curcumin caused a significant reduction of MMP1 (Figure 5v) and MMP3 (Figure 5b) at 10 μM and 20 μM. For MMP13, all concentrations of curcumin caused a significant reduction (Figure 5c). Expression of IL-1β (Figure 5d) and IL-6 (Figure 5e) was significantly inhibited at both, 10 μM and 20 μM, while the lowest concentration caused a slight increase of IL-6. IL-8 expression was also decreased at 20 μM (Figure 5f). In contrast, TNF-α expression was significantly increased at all three curcumin concentrations, with the most prominent effects at 20 μM (Figure 5g). Furthermore, TNF-α expression was also increased upon curcumin treatment alone (i.e. if no IL-1β pretreatment was performed), while all other target genes remained unaltered under these conditions (data not shown). TLR2 expression was significantly reduced with each concentration (Figure 5h). For summarized values see Additional file 4: Table 2.

### Analysis of NF-κB

**Figure 6:**
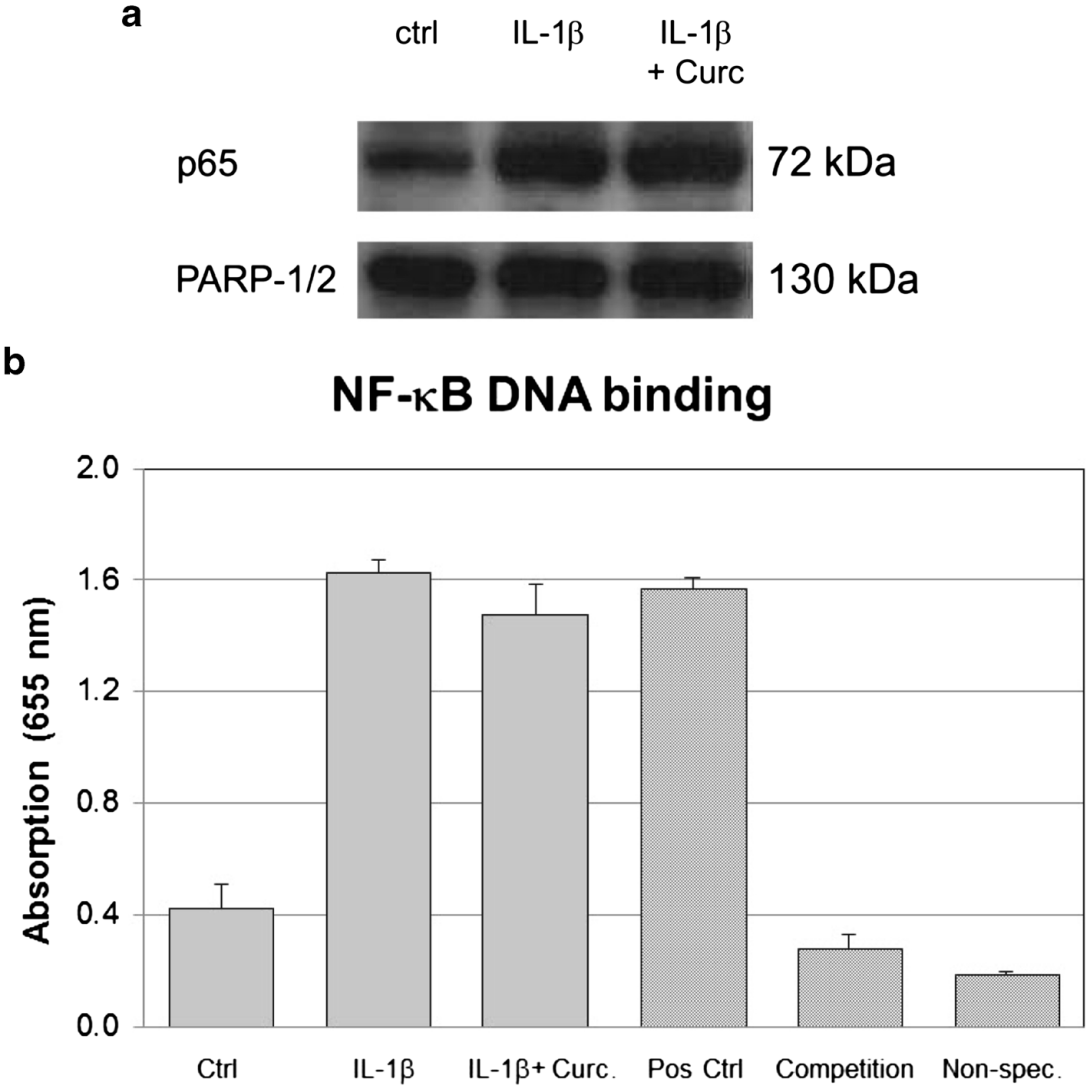
**Effects of curcumin on nuclear translocation of p65/NF-κB, measured by immunoblotting of p65 (n = 3, nuclear extracts, 60 min).** 6a: Nuclear translocation of p65, measured by immunoblotting (n=3, nuclear extracts, 60 min). One representative sample is shown and PARP-1/2 is used as a loading control. 6b: NF-kB DNA binding activity, measured by NF-kB transcription factor assay (n=2, nuclear extracts, 60 min: without statistical evaluation). Note that different internal controls (positive control, competition control and unspecific binding control) were used to validate the test.

Immunoblotting of p65 in nuclear extracts of untreated, IL-1β-treated and IL-1β/curcumin-treated cells revealed that IL-1β-treatment caused nuclear translocation of p65 after 60 min. However, compared to IL-1β stimulated samples, curcumin treatment did not reduce levels of the target protein in nuclear extracts (equal protein loading was confirmed by PARP detection) (Figure 6a). Using the NF-κB/p65 transcription factor assay, we provide further evidence that IL-1β strongly induced NF-κB DNA binding (similar to the positive control =  Pos Ctrl), while curcumin was not able to reduce levels after IL-1β stimulation (Figure 6b). Internal assay controls (i.e. competition and non-specific binding control) ensured validity of the test.

### Analysis of MAP kinases (p38, ERK, JNK)

**Figure 7:**
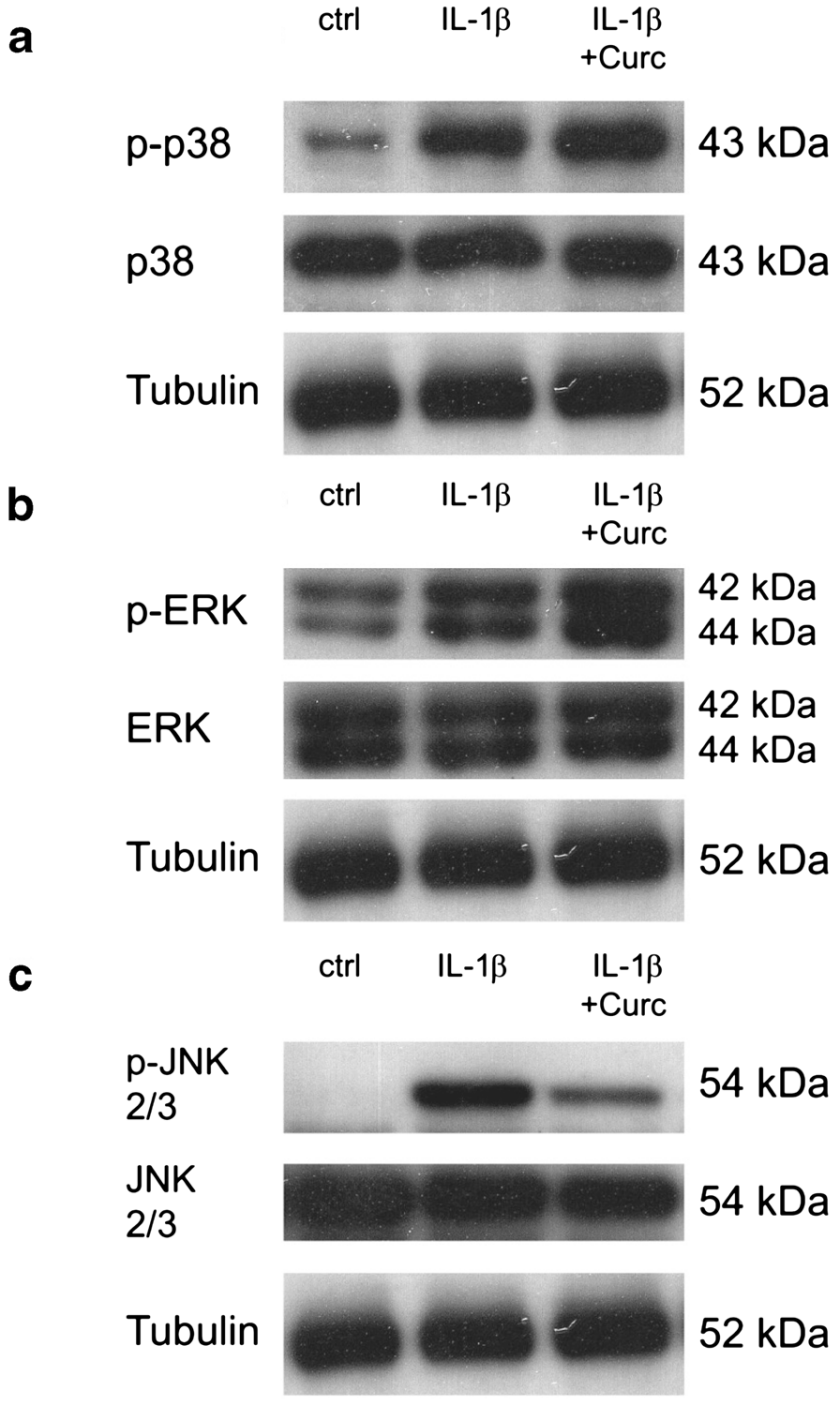
**Effects of curcumin on MAP kinase activity, measured by immunoblotting of p38/p-p38 (7a), ERK/p-ERK (7b) and JNK/p-JNK (7c) (n = 5, whole cell extracts, 15 min).** One representative sample is shown and tubulin is used as a loading control.

Effects of curcumin on MAP kinase activity were investigated by detection of levels of phosphorylated and unphosporylated p38 (Figure 7a), ERK (Figure 7b) and JNK (Figure 7c) using immunoblotting technique of whole cell extracts. Results demonstrate that IL-1β treatment increased levels of phosporylated p38, ERK and JNK after 15 min, which is indicative of activation of these MAP kinases. Treatment with curcumin reduced activity of JNK compared to IL-1β treatment (i.e. reduced levels of p-JNK), but further increased levels of p-ERK and p-p38 compared to IL-1β treatment. Levels of unphosporylated p38, ERK and JNK were similar in all groups. Equal protein loading was confirmed by tubulin detection.

## Discussion

### Changes in gene expression

Curcuma is not only an ancient spice, but also a traditional remedy that has been used in Indian and Chinese medicine to treat indigestion and many other medical issues. Since the 1970s, the anti-inflammatory compounds called curcuminoids were discovered in the spice, with one (and probably the most important component) being curucmin [22]. Because of its anti-inflammatory properties, curcuma and its components (especially curcumin) have been investigated in osteoarthritis and rheumatoid arthritis during the past one to two decades, while only one paper has been published on the effects of curcumin on intervertebral disc cells so far [23].

Our results clearly show that the different curcuma extracts influenced cellular behavior in a different manner. While the curcuma EtOH extract (which contained only little amounts of curcumin, as shown by HPLC/MS) had no effect and was thus considered to be not suitable for further investigations, the curcuma DMSO extract as well as the DMSO-soluble compound curcumin (which was shown to be present in the DMSO extract by HPLC/MS) reduced levels of some disc-specific, major proinflammatory cytokines and matrix degrading enzymes in our *in vitro* experiments. We were able to demonstrate that the observed effect was not due to the biologic activity of the solvents DMSO and EtOH (Additional file 1: Figure 3 and Additional file 2: Figure 4), although the anti-inflammatory properties of DMSO have most recently been described in human intestinal cells [24].

Specifically, we could demonstrate a reduction in gene expression of IL-6, MMP1, MMP3 and MMP13 when treating IL-1β prestimulated cells with the curcuma DMSO extract. Additionally, IL-1β and IL-8 were reduced by curcumin treatment after 6 hours. As effects were comparable between the curcuma DMSO extract and curcumin and as curcumin was detected at high concentrations in the DMSO extract by HPLC/MS, we hypothesize that the major bioactive substance in curcuma DMSO extracts acting on human intervertebral disc cells could be curcumin. Due to the beneficial effects of curcumin, this natural compound may be of benefit for patients with inflammation-related back pain, with the potential mode of application being intradiscal injection. Albeit curcumin is well known for its low bioavailability in case of oral consumption, *in vivo* concentrations after injections should not be a limiting factor.

The observed gene expression results are similar to effects that were observed when treating other cell types with curcumin, e.g. leading to a reduction in IL-1β [25-28], IL-6 [25,28-30], IL-8 [25,31], MMP1 [32], MMP3 [26,32,33] and MMP13 [32,34]. For IL-6, we observed a slight increase at the lowest concentrations (1.5 fold), but a decrease at higher concentrations. This may be due to biphasic effects of curcumin that are based on its dual function to either scavenge or produce reactive oxygen species [35]. However, the biphasic nature of curcumin cannot explain that higher concentrations of curcumin strongly stimulated expression of TNF-α in human intervertebral disc cells (both, without pretreatment as well as after IL-1β prestimulation), which is different from what is described in the literature [25,28,36]. Based on the current study we do not know whether this effect would also occur on the protein level and *in vivo*. Therefore, further studies are thus required to demonstrate safety and usefulness of curcumin in human application.

So far, only one study investigated the effect of curcumin on human intervertebral disc cells [23]. This study tested curcumin for its effects on matrix protein gene expression, but not on the expression of proinflammatory cytokines or matrix degrading enzymes. Results of Yu *et al.*’s study indicated that curcumin is also able to attenuate an IL-1 induced inhibition of SOX9 and collagen–II expression at 20 μg/ml (= 54.3 μM), which is higher than the concentrations used in the present study and which was shown to be a damaging concentration for other (disc-related) cell types (e.g. C-28/I2 = a chondrocyte cell line) [37]. Furthermore, it has to be noted that both, Yu’s as well as our study were performed in a 2D culture system, which can cause certain phenotypic changes of disc cells and may thus possibly influence cellular behavior to the tested treatment.

### Pathway analysis

Curcumin seems to exhibit its anti-inflammatory and anti-catabolic effects through versatile mechanisms. So far, in different cell types, mainly inhibition of NF-κB, MAP kinases and Toll-like receptors have been shown to play a role [31,38-41].

#### NF-κB

Inhibition of the transcription factor NF-κB is the best described mechanism of action of curcumin in the literature [42]. A recent study in chondrocytes showed that curcumin inhibits phosphorylation and degradation of IκBα (= nuclear factor of kappa light polypeptide gene enhancer in B-cells inhibitor, alpha) and thus translocation of the p65 subunit of NF-κB to the nucleus, indicating that inhibition of the NF-κB pathway takes place at a step before IκBα phosphorylation [41]. In intestinal epithelial cells, curcumin seems to exert its effects by blocking a signal leading to IKK (= IκB kinase) activity [31]. However, in our experimental setting, curcumin did not seem to reduce IL-1β induced nuclear translocation of NF-κB/p65 or NF-κB DNA binding, which is in contrast to data obtained by Yu *et al*. on intervertebral disc cells [23].

#### Toll-like receptors

We were able to demonstrate a down-regulation of TLR2 mRNA expression after treating IL-1β prestimulated IVD cells with curcumin, which confirms findings in other cell types such as monocytic THP-1 cells, HL-60 promyelocytic leukemia cells and primary peripheral blood polymorphonuclear neutrophils [38]. However, in a leukemia cell line, Reuter *et al*. showed an increase in TLR2 due to curcumin, although most inflammatory mediators were simultaneously down-regulated in this study [25]. There is also some evidence in the literature that curcumin can reduce expression levels of TLR4 [29,43]. Based on how little is known about TLRs and curcumin so far, more research is needed to establish a causal relationship between therapeutic efficacy of curcumin and TLR2 activity.

#### MAP kinases

The mitogen-activated protein (MAP) kinase signaling pathways, including JNK, p38 and extracellular-signal regulated kinase (ERK), play an important role in the regulation of inflammatory responses [14]. As MAP kinases are regulated by phosphorylation cascades, their activity can be determined by detecting phosphorylation levels. We found that curcumin was able to inhibit phosphorylation of JNK in IL-1β prestimulated IVD cells, which is similar to primary chondrocytes [34,44]. Importantly, pharmacological inhibition of JNK (by SP600125) has previously been shown to suppress MMP1, MMP3 and MMP13 mRNA expression in bovine and murine IVD cells [45,46] (reviewed in [47]).

In contrast, phosphorylation of p38 and ERK was induced upon curcumin treatment in IL-1β prestimulated IVD cells as well as in curcumin-only treated IVD cells, with a synergistic effect of IL-1β and curcumin [48-50]. It may be possible that activation of p38 and ERK led to the up-regulation of TNF-α expression which was observed when IL-1β pretreated and untreated IVD cells were exposed to curcumin, but our experimental design does not allow to establish a causal relationship between MAP kinase activation and TNF-α expression. Furthermore, activation of ERK and p38 does not only control inflammation, but also several other cellular functions, such as cell cycle progression for ERK (i.e. transition from the G1 to the S phase of the cell cycle) [51] and cell growth and differentiation for p38 [52], indicating that MAP kinase related cellular control is of high complexity.

## Conclusion

In conclusion, the results of this study demonstrate that curcumin may become an attractive alternative for the treatment of discogenic back pain when envisaging an intradiscal injection method, which will circumvent the low bioavailability of curcumin. Although we were able to show in a previous study (using a similar experimental set-up) that another anti-inflammatory substance (resveratrol), caused pain-reducing effects in a rodent model of radiculopathic pain *in vivo*[53], the analgetic effect of curcumin first needs to be confirmed before clinical trials are reasonable.

## Misc

Marina Klawitter and Lilian Quero contributed equally to the manuscript.

## Competing interests

All authors declare that they have no competing interests.

## Authors’ contributions

MK carried out the cell culture experiments, performed statistical analysis and helped to draft the manuscript. LQ carried out the cell culture experiments, performed statistical analysis and helped to draft the manuscript. JK participated in the design of the study, provided clinical sample and medical scientific input and helped to draft the manuscript. AG designed and carried out the HPLC/MS experiments together with BK and helped to draft the manuscript. BK designed and carried out the HPLC/MS experiments together with AG and helped to draft the manuscript. OH participated in the design of the study, provided clinical sample and medical scientific input and helped to draft the manuscript. NB participated in the design of the study, conceived funding for the study and helped to draft the manuscript. KW conceived funding of the study, designed and coordinated the study, performed statistical analysis and drafted the manuscript. All authors read and approved the final manuscript.

## Supplementary Material

Additional file 1 **Figure S1.** Effects of 0.03% DMSO on mRNA levels of candidate genes after 6 hours, indicated as fold change relative to DMSO-free (i.e. untreated) controls (set to 1). Data was obtained by real-time RT-PCR (2^-ΔΔCt^ method) and is presented as Mean and SEM (n = 3). Each gene was normalized to its respective DMSO-free control, which was always set to 1 (only one exemplary untreated control bar is shown). Click here for file

Additional file 3 **Table S3.** Summarized values of the graphical illustration of the effects of the curcuma DMSO and EtOH extracts shown in Figure 1**.** Quantitative values of the anti-catabolic and anti-inflammatory effects of the curcuma DMSO and EtOH extracts on mRNA levels of candidate genes after 6 hours (indicated as fold change relative to IL-1β-prestimulation: 100%) are given only if a ***statistically significant reduction*** occurred (p < 0.05). Note that IL-1β prestimulated cells also contain 0.03% of DMSO or EtOH respectively. Data was obtained by real-time RT-PCR (2^-ΔΔCt^ method) and is presented as Mean and SEM (n = 5). Click here for file

Additional file 2 **Figure S2.** Effects of 0.03% EtOH on mRNA levels of candidate genes after 6 hours, indicated as fold change relative to EtOH-free (i.e. untreated) controls (set to 1). Data was obtained by real-time RT-PCR (2^-ΔΔCt^ method) and is presented as Mean and SEM (n = 3). Each gene was normalized to its respective EtOH-free control, which was always set to 1 (only one exemplary untreated control bar is shown). Click here for file

Additional file 4 **Table S4.** Summarized values of the graphical illustration of the effects of curcumin shown in Figure 5. Quantitative values of the anti-catabolic and anti-inflammatory effects of curcumin on mRNA levels of candidate genes after 6 hours (indicated as fold change relative to IL-1β-prestimulation: 100%) are given only if a ***statistically significant reduction*** occurred (p < 0.05). Note that IL-1β prestimulated cells also contain 0.03% of DMSO. Data was obtained by real-time RT-PCR (2^-ΔΔCt^ method) and is presented as Mean and SEM (n = 5). Click here for file
