# Supplementary figures and images for: Curcuma DMSO extracts and curcumin exhibit an anti-inflammatory and anti-catabolic effect on human intervertebral disc cells, possibly by influencing TLR2 expression and JNK activity
Source: J Inflamm (Lond). 2012 Aug 21;9:29. doi: 10.1186/1476-9255-9-29 (PMC3506446; doi:10.1186/1476-9255-9-29)

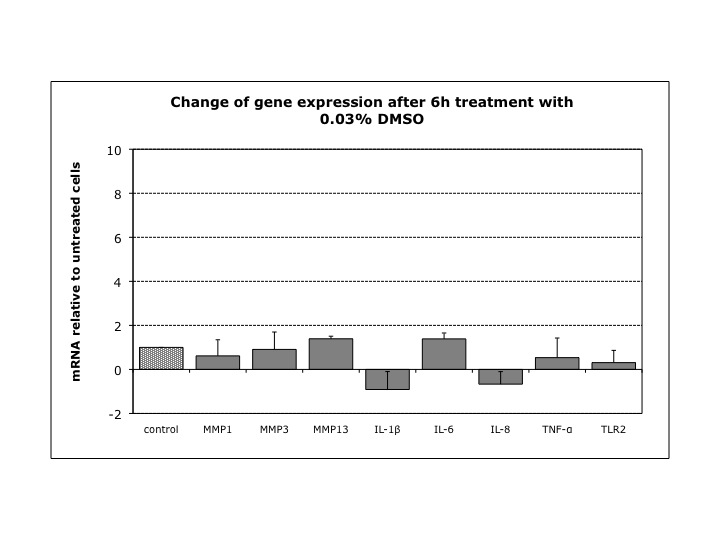

Supplement: Additional file 1 — Figure S1. Effects of 0.03% DMSO on mRNA levels of candidate genes after 6 hours, indicated as fold change relative to DMSO-free (i.e. untreated) controls (set to 1). Data was obtained by real-time RT-PCR (2-ΔΔCt method) and is presented as Mean and SEM (n = 3). Each gene was normalized to its respective DMSO-free control, which was always set to 1 (only one exemplary untreated control bar is shown). [file 1476-9255-9-29-S1.jpeg]

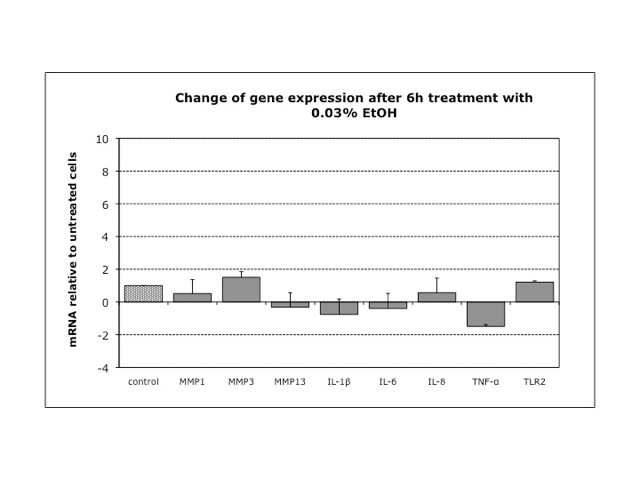

Supplement: Additional file 2 — Figure S2. Effects of 0.03% EtOH on mRNA levels of candidate genes after 6 hours, indicated as fold change relative to EtOH-free (i.e. untreated) controls (set to 1). Data was obtained by real-time RT-PCR (2-ΔΔCt method) and is presented as Mean and SEM (n = 3). Each gene was normalized to its respective EtOH-free control, which was always set to 1 (only one exemplary untreated control bar is shown). [file 1476-9255-9-29-S2.jpeg]
