# Supplementary material for: Curcuma DMSO extracts and curcumin exhibit an anti-inflammatory and anti-catabolic effect on human intervertebral disc cells, possibly by influencing TLR2 expression and JNK activity
Source: J Inflamm (Lond). 2012 Aug 21;9:29. doi: 10.1186/1476-9255-9-29 (PMC3506446; doi:10.1186/1476-9255-9-29)
Supplement: Additional file 4 — Table S4. Summarized values of the graphical illustration of the effects of curcumin shown in Figure 5. Quantitative values of the anti-catabolic and anti-inflammatory effects of curcumin on mRNA levels of candidate genes after 6 hours (indicated as fold change relative to IL-1β-prestimulation: 100%) are given only if a statistically significant reduction occurred (p < 0.05). Note that IL-1β prestimulated cells also contain 0.03% of DMSO. Data was obtained by real-time RT-PCR (2-ΔΔCt method) and is presented as Mean and SEM (n = 5). [file 1476-9255-9-29-S4.doc]

**Supplementary Data**

**Table B**. **Summarized values of the graphical illustration of the effects of curcumin shown in Figure 5.** Quantitative values of the anti-catabolic and anti-inflammatory effects of curcumin on mRNA levels of candidate genes after 6 hours (indicated as fold change relative to IL-1β-prestimulation: 100%) are given only if a ***statistically significant reduction*** occurred (p < 0.05). Note that IL-1β prestimulated cells also contain 0.03% of DMSO. Data was obtained by real-time RT-PCR (2^-ΔΔCt^ method) and is presented as Mean and SEM (n=5).

| Gene | Curcumin  5 µm | Curcumin  10 µm | Curcumin  20 µm |
| --- | --- | --- | --- |
| IL-1β | ↔ | -70% | -75% |
| IL-6 | ↑ | -44% | -80% |
| IL-8 | ↔ | ↔ | -40% |
| MMP1 | ↔ | -60% | -77% |
| MMP3 | ↔ | -66% | -71% |
| MMP13 | -52% | -87% | -85% |
| TNF-α | ↑ | ↑ | ↑ |
| TLR2 | -39% | -72% | -80% |
